# Improving Implementation of Fertility Preservation Benefit Mandates

**DOI:** 10.1001/jamahealthforum.2025.3166

**Published:** 2025-09-12

**Authors:** Sara B. McMenamin, Bonnie N. Kaiser, Ricardo E. Flores Ortega, Sara W. Yoeun, Melina A. Economou, Natasha Bisarya, Kara N. Goldman, Jennifer Levine, Glenn L. Schattman, Gregory A. Aarons, Sally A. D. Romero, H. Irene Su

**Affiliations:** 1 Herbert Wertheim School of Public Health and Human Longevity Science, University of California, San Diego, La Jolla; 2 Department of Anthropology and Global Health Program, University of California, San Diego, La Jolla; 3 Moores Cancer Center, University of California, San Diego, La Jolla; 4 Northwestern University Feinberg School of Medicine, Chicago, Illinois; 5 Division of Pediatric Oncology, Children’s National Medical Center, Washington, DC; 6 Ronald O. Perelman and Claudia Cohen Center for Reproductive Medicine, Weill Medical College of Cornell University, New York, New York; 7 Department of Psychiatry, University of California, San Diego, La Jolla; 8 Department of Obstetrics, Gynecology and Reproductive Sciences, University of California, San Diego, La Jolla; 9 University of California, San Diego ACTRI Dissemination and Implementation Science Center, La Jolla

## Abstract

**Importance:**

More than 90 000 adolescents and young adults are diagnosed with cancer and exposed to treatments that may threaten their future fertility every year. Fertility preservation (FP) services, such as egg, embryo, and sperm freezing, are available to preserve future fertility but are often underused by patients in part due to high cost. Eighteen states and Washington, DC, recently began mandating health insurance coverage for FP services, yet patients and clinicians report difficulty accessing mandated services.

**Objective:**

To systematically identify determinants of implementing FP benefit mandates in fertility and oncology clinics to inform intervention development and future public policy.

**Design, Setting, and Participants:**

In this mixed-methods study, fertility and oncology clinics from California, Illinois, and New York were selected from the 8 states that had FP benefit mandates in place in 2020 to maximize diversity of the state-level characteristics that may impact mandate implementation. Fertility and oncology clinic representatives (health care clinicians, financial counselors, and other administrative personnel) identified as being the most knowledgeable regarding implementation of FP benefit mandates were interviewed or surveyed using the Exploration, Preparation, Implementation, Sustainment (EPIS) framework. Interviews and surveys were conducted from July 2020 to November 2023, and data were analyzed from September 2022 to June 2024.

**Exposure:**

Passage of a state-level FP benefit mandate.

**Main Outcomes and Measures:**

Barriers and facilitators to implementing access to FP benefits after mandate passage at clinic and patient levels.

**Results:**

This study included 48 participants from 24 oncology and fertility clinics and 2 fertility pharmacies. Interviews (n = 48) and surveys (n = 17) were used to identify determinants of successful FP benefit mandate implementation. The top 3 barriers identified included (1) time-consuming nature of interactions between clinics and insurers, (2) patients’ lack of knowledge on their benefits and how to find them, and (3) holes in coverage and heterogeneity of benefits across health insurance plans. The top 3 facilitators included (1) dedicated clinic financial counselors who guide patients on benefit verification, (2) clearly defined FP benefits in member handbooks, and (3) health insurance plan preexisting in vitro fertilization insurance benefit.

**Conclusions and Relevance:**

In this study, multiple barriers and facilitators were systematically identified to assist in improving implementation of FP benefit mandates. These findings support policies and intervention development for FP patients and clinics, and future legislative and regulatory efforts aimed at increasing timely access to FP insurance benefits.

## Key Points

**Question:**

What barriers and facilitators impact access to fertility preservation services following fertility preservation insurance benefit mandate passage?

**Findings:**

In this mixed-methods study including 48 fertility and oncology clinic representatives across 26 institutions in 3 states, fertility and oncology clinics assisted patients accessing insurance for fertility preservation procedures after mandate passage. Clinic-insurer and patient-insurer communication on eligibility for mandated benefits and covered services were barriers across clinics, while facilitators included clinic commitment to financial navigation resources and insurers with clear benefit structure and member handbooks.

**Meaning:**

Interventions and tools to address barriers and maximize facilitators within and between insurers, clinics, and patients are needed to improve access to fertility preservation services.

## Teaser

This mixed-methods study identifies determinants of implementing fertility preservation benefit mandates in fertility and oncology clinics.

## Introduction

Approximately 5% of all US cancers (90 000) are diagnosed among people aged 15 to 39 years.^1,2^ Many cancer treatments can increase the likelihood of infertility.^3,4^ The World Health Organization defines infertility as a disease, and fertility preservation (FP) treatments, such as egg, sperm, or embryo freezing, are effective in preventing this disease.^5^ Expensive and similar to infertility treatments, these services are not commonly covered by health insurance, with an average egg freezing cycle costing $10 000 to $15 000 and required up-front cash payment.^6^ Even with coverage, FP treatments require finding in-network fertility specialists, assisted reproductive technology laboratories, and sperm banks, many of which do not traditionally contract with health insurance plans. As oncologists are sometimes hesitant to discuss FP options with patients about to undergo cancer treatment if they perceive that they cannot afford or access them, patients often lack the information needed to make decisions regarding their treatment options leading to information asymmetry.^7^

To address financial barriers to accessing FP, states began passing legislation requiring health insurance plans to cover FP services (benefit mandates). Currently, 18 states and Washington, DC, have benefit mandates, and 19 states are either actively or previously considered such legislation.^8^ Initial studies suggest modest increases in FP counseling and service utilization in states with mandates.^7,9,10^ Prior research documenting the implementation of California’s FP benefit mandate found that clinicians and patients report difficulty accessing mandated benefits.^11^ A gap in knowledge remains on both the range of barriers to accessing mandated benefits and the experience across multiple states with FP mandates.

To reach patients, FP benefit mandates are implemented at multiple levels as defined by the implementation science Exploration, Preparation, Implementation, Sustainment (EPIS) framework. This includes oncology and fertility clinics and patients in the inner context and state health insurance regulators and health insurers in the outer context (Figure).^11,12^ Barriers at any level (legislation, regulation, insurer, or clinic) can prevent FP benefits from reaching patients.^11,13^ Our objective is to systematically identify determinants (ie, barriers and facilitators) to implementing access to mandated FP benefits in fertility and oncology clinics to inform intervention development and future public policy.

**Figure.**
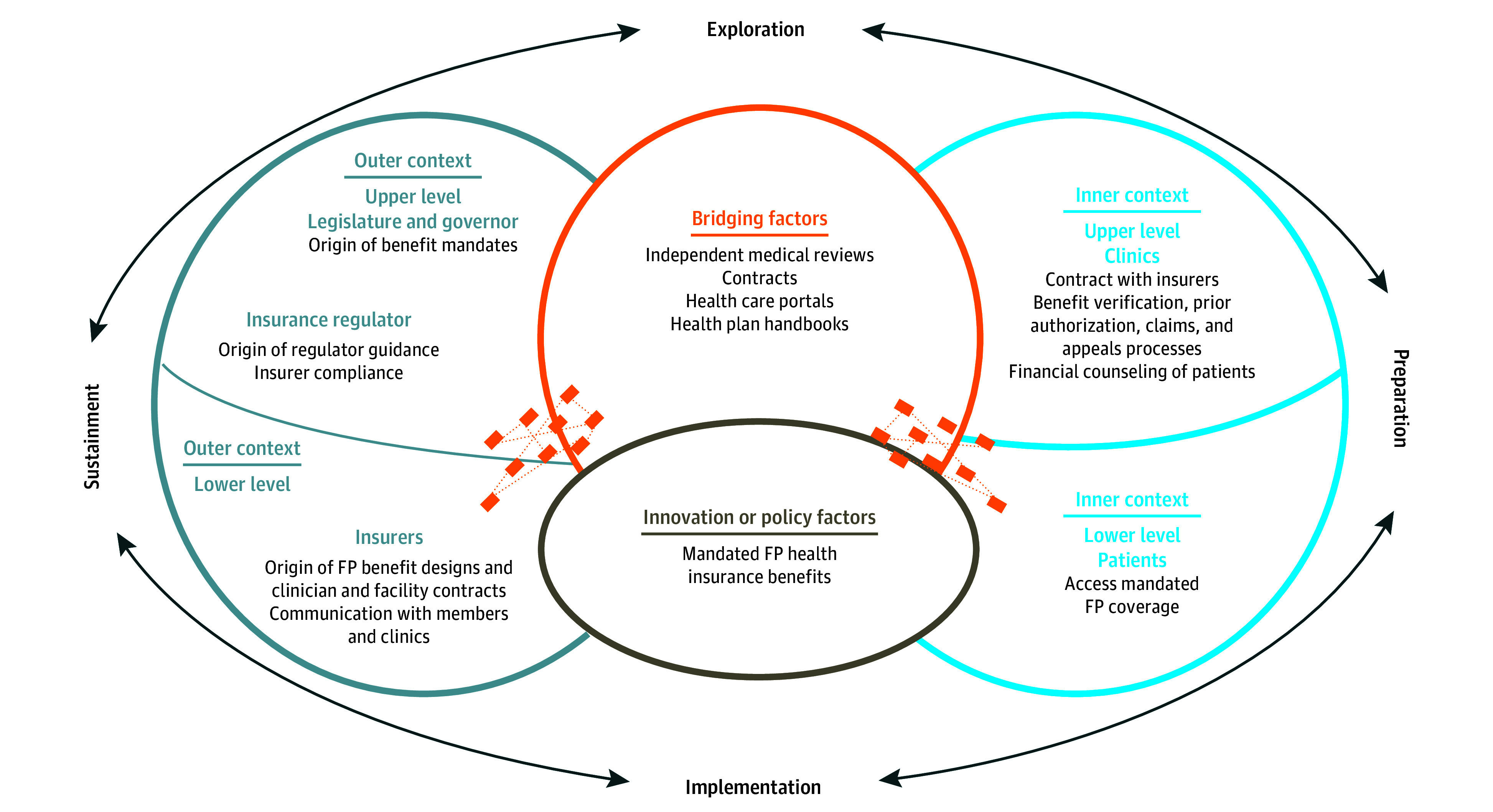
Exploration, Preparation, Implementation, Sustainment (EPIS) Framework Demonstrating Fertility Preservation (FP) Benefit Mandate Implementation Process Adapted from Crable et al,^12^ which was published open access. The orange dashes represent how the bridging factors bridge the inner and outer contexts at the lower and upper levels.

## Methods

### Study Design

We conducted semistructured interviews and follow-up web-based surveys with representatives from fertility and oncology clinics and fertility pharmacies. The interview guide (Table 1) was developed using implementation science frameworks optimized for policy implementation, including EPIS, the Consolidated Framework for Implementation Research, and the Theoretical Framework for Policy Implementation.^12,14,15^ These frameworks are useful for understanding system-level drivers of policy implementation, both internal and external to clinics. Respondents provided written informed consent for the study, consented verbally to the interview and recordings, and were compensated $20 for their time. The study was approved by the University of California, San Diego Institutional Review Board. Data were collected and reported in accordance with the Standards for Reporting Qualitative Research (SRQR) reporting guideline.

**Table 1.** Semistructured Interview Guide^a^

Question	Related construct
**Clinician questions**	
1. How were you made aware of the state FP benefit law?	Inner context: access to knowledge
2. What processes in your clinic changed to help patients access these FP insurance benefits?	Implementation: tailoring, adapting
3. What was your role in your clinic’s implementation of access to FP insurance benefits?	Individuals: role
4. Does your clinic assess the effectiveness of implementation of access to FP benefits? If so, how?	Process reflecting/evaluating
5. How much variation have you observed in FP insurance benefits among your patients?	Innovation: design
6. How does FP insurance coverage for an individual patient affect your clinical recommendations to patients who need FP services?	Service outcome
7. How has the FP benefit mandate impacted your patients’ access to FP services?	Service outcome
8. How have you interacted with insurers on FP benefits?	Bridging
9. How would a patient interact with the clinic to access FP benefits?	Bridging
10. How could these processes be improved?	Innovation: adaptability
11. What barriers do you think patients now face in accessing FP benefits?	Implementation: assessing needs
12. What types of training or support materials do you think the clinic team or patients need to access benefits?	Implementation: assessing needs
13. Who else do you think we should speak with to understand mandate implementation? Would you be able to facilitate introductions?	NA
**Administrative staff questions**	
1. What did your clinic do to implement access to FP health insurance benefits?	Implementation: tailoring, adapting
2. How did financial counseling change because of the mandate?	Implementation: tailoring, adapting
3. How did benefit verification change because of the mandate?	Implementation: tailoring, adapting
4. How did claims and appeals change because of the mandate?	Implementation: tailoring, adapting
5. How did the clinic’s insurance contracts with insurers change because of the mandate?	Implementation: tailoring, adapting
6. What is your role in your clinic’s implementation of access to the FP insurance benefits?	Individual: role
7. In the clinic, how does the financial team learn about new contracted FP benefits?	Inner context: access to knowledge
8. What factors about the FP benefits make it more or less difficult to implement in the clinic compared with infertility benefits?	Innovation: complexity, design
9. How do you help patients access FP benefits?	Individuals: role
10. What educational or support resources do you provide or do you think patients need to access FP benefits?	Implementation: assessing needs
11. How variable are FP benefits across plans?	Innovation: design
12. How variable are out-of-pocket costs for patients with FP benefits?	Innovation: design
13. How variable is the accessibility of FP benefits across commercial insurance plans (for Illinois, between commercial and Medicaid plans)?	Innovation: design
14. How has accessing FP benefits been similar to or different from non-FP benefits (eg, infertility)?	Implementation: outcome
15. How do you assess effectiveness of clinic services for helping patients access FP benefits?	Service: outcome
16. Overall, how has the FP benefit mandate impacted patient access to FP services?	Service: outcome
17. Who else do you think we should speak with to understand mandate implementation by insurers and clinics? Would you be able to facilitate introductions?	NA

Abbreviations: FP, fertility preservation; NA, not applicable.

^a^ Interview guide adapted from the Exploration, Preparation, Implementation, Sustainment framework,^12^ Consolidated Framework for Implementation Research,^14^ and Theoretical Framework for Policy Implementation.^15^

### Recruitment

Fertility and oncology clinic representatives were recruited from 3 states: California, Illinois, and New York. States were purposively selected from the 8 that had FP benefit mandates at study initiation (2020) to maximize diversity of state-level characteristics that may impact implementation including: (1) mandate population, (2) preexisting in vitro fertilization mandate, and (3) level of specificity of mandate language and regulator guidance (eTable 1 in Supplement 1).^13^ Within selected states, recruitment began at high-volume FP clinics with research and clinical expertise, followed by network-based sampling.^16^ Within each clinic, we interviewed staff most familiar with mandate implementation, typically a clinician, financial counselor, and/or other administrative personnel. Recruitment was conducted in 3 waves, with analysis and iterative refining of the interview guide between waves. Participants were recruited until data saturation was reached (ie, no new themes emerging).

### Data Collection and Procedure

Between July 2020 and November 2023, 1-hour interviews to document the FP benefit mandate implementation process were conducted, recorded via Zoom (Zoom Communications) and transcribed using Otter.ai. Through analysis of transcripts, we identified modifiable barriers and facilitators. Participants were invited to complete a web-based survey, first rating the relevance of the 11 barriers and 15 facilitators on a scale from 1 to 4. They were then asked to rank these same items against each other to assess their relative harmfulness (barrier) or helpfulness (facilitator) for accessing FP benefits. This process required each item to receive a distinct ranking, forcing respondents to prioritize among the items. Because modifiable barriers and facilitators were not directly in the scope of oncology practice or fertility pharmacies, we only recruited survey participants from fertility clinic interviewees.

### Statistical Analysis

Data were analyzed using MAXQDA software version 2022 (VERBI GmbH). Thematic analysis was used. Deductive codes were developed based on implementation science frameworks and inductive codes were identified through team-based review of a subset of transcripts.^14,15^ Following an intercoder agreement process to refine the codebook and establish reliability, each transcript was coded by 2 of 3 coders (R.F.O., S.W.Y., M.A.E.), with disagreements resolved by consensus. Code summaries were prepared, including barriers and facilitators summaries. Descriptive statistics were used to summarize relative ratings and rankings of identified barriers and facilitators. To assess impact of oversampling California clinics, sensitivity analysis excluding California clinics was conducted (eTables 2 and 3 in Supplement 1). Data were analyzed from September 2022 to June 2024.

## Results

### Sample Characteristics

This study included 48 participants from 23 oncology and fertility clinics and 2 fertility pharmacies, including 13 fertility clinicians, 9 oncology clinicians, 5 pharmacy staff, 7 financial navigators, 8 financial counselors, and 6 clinic administrators. Most clinics were in California (15 [65%]), followed by Illinois (5 [22%]), and New York (3 [13%]). While equal numbers of interviews across states were planned, data collection was stopped after reaching saturation and confirming that barriers and facilitators were similar across states. Among 25 interview participants from fertility clinics, 17 also completed the follow-up survey (California, 7 [41%]; Illinois, 7 [41%]; New York, 3 [18%]). Barriers and facilitators were categorized at the insurer, clinic, and patient levels (Table 2 and Table 3). All determinants were rated as moderately to very relevant to accessing FP benefits, confirming interview findings.

**Table 2.** Identified Barriers to Fertility Preservation (FP) Services and Stakeholder Ranking of Perceived Level of Harm by Health System Level^a^

Barrier to FP services	Perceived level of harm	
	Rating, mean (SD)^b^	Ranking^b^
Insurer-related factors		
Time-consuming member services interactions	3.5 (0.73)	1
FP benefits: coverage holes and heterogeneity across plans	3.3 (0.70)	3
Confusion between FP and infertility services	3.5 (0.63)	6
Challenging benefit verification process	3.0 (0.82)	7
Issues with FP services diagnostic and *Current Procedural Terminology* codes	3.3 (0.77)	8
Insufficient clinician and facility networks	3.3 (0.79)	10
Ineffective insurer website clinician search tools	3.3 (0.86)	11
Clinic-related factors		
Frequent turnover of clinic financial staff	3.5 (0.82)	5
Patient-related factors		
Patient lacks knowledge of health plan handbook and benefits	3.3 (0.79)	2
Issues with accessing financial resources	2.9 (0.89)	9
Policy (ie, benefit mandate) related factors		
State benefit mandate does not apply to all patients	3.2 (0.91)	4

^a^ Health system levels are mapped to the following Exploration, Preparation, Implementation, Sustainment constructs: insurer (lower-level, outer context); clinic (upper-level, inner context), patient (lower-level, inner context), and policy (upper-level, outer context).

^b^ Respondents were first asked to rate the perceived level of harm of each of the factors identified in the interviews using a 4-point rating scale, with 1 indicating not at all harmful, 2 indicating a little harmful, 3 indicating moderately harmful, and 4 indicating very harmful. Respondents were next asked to prioritize the harmfulness of each item by ranking it relative to the others. This process required each item to receive a distinct, unique ranking, forcing respondents to prioritize among the items. For the ranking scale, the lower number is ranked as more harmful. There were 17 respondents, including 7 clinic administrators, 6 clinicians, and 4 financial counselors.

**Table 3.** Identified Facilitators to Fertility Preservation (FP) Services and Stakeholder Ranking of Perceived Level of Helpfulness by Health System Level^a^

Facilitator to FP services	Perceived level of helpfulness	
	Rating, mean (SD)^b^	Ranking^b^
Insurer-related factors		
FP benefit is clearly described in health plan handbook	3.6 (0.92)	2
Preexisting in vitro fertilization insurance benefit for infertility	3.7 (0.75)	3
Clinic-related factors		
Clinic financial counselor		
Guides patients on benefit verification	3.7 (0.59)	1
Expertise to escalate to insurer supervisors	3.8 (0.59)	5
Has contact at health insurer	4.0 (0.0)	4
Expertise on benefit verification processes	3.8 (0.43)	6
Is a dedicated staff position	3.9 (0.24)	7
Educates insurers about FP benefit mandate	3.2 (1.2)	8
List of fertility preservation diagnosis and *Current Procedural Terminology* codes	3.7 (0.69)	10
Templated letter of medical necessity for fertility preservation	3.5 (0.86)	11
Templated appeal documents for FP services mentions mandate	3.4 (0.92)	14
Patient-related factors		
Patient’s employer human resources department is knowledgeable about FP	3.8 (0.55)	9
Patient is educated on how to submit appeals for FP services	3.6 (0.70)	12
Patient is guided on accessing loans and philanthropic resources for FP	3.5 (0.79)	13
Patient has materials on FP service costs	3.5 (0.71)	15

^a^ Health system levels are mapped to the following Exploration, Preparation, Implementation, Sustainment constructs: insurer (lower-level, outer context); clinic (upper-level, inner context), patient (lower-level, inner context), and policy (upper-level, outer context).

^b^ Respondents were first asked to rate the perceived level of helpfulness of each of the factors identified in the interviews using a 4-point rating scale, with 1 indicating not at all helpful, 2 indicating a little helpful, 3 indicating moderately helpful, and 4 indicating very helpful. Respondents were next asked to prioritize the helpfulness of each item by ranking it relative to the others. This process required each item to receive a distinct, unique ranking, forcing respondents to prioritize among the items. For the ranking scale, the lower number is ranked as more helpful. There were 17 respondents, including 7 clinic administrators, 6 clinicians, and 4 financial counselors.

### Barriers

#### Mandate Design

One highly ranked barrier was that mandates do not apply to the entire insured population. State mandates do not apply to self-funded employer groups (which are subject to federal regulation) or coverage from an employer headquartered out of state. This makes it difficult to determine whether patients are eligible for mandated coverage. One clinic administrator in Illinois reported that “most people have no idea if [their] company has a self-funded policy.” This results in clinic staff and patients having to undergo the time-consuming process of contacting health insurers and employer human resources to verify coverage.

#### Insurer Level

Participants indicated that most barriers were at the insurer level. The top-ranked barrier was time-consuming interactions between clinics and insurers to verify coverage, submit prior authorization, and submit appeals for coverage denials. One clinician in New York described that “our insurance and billing people spend lots of time on the phone, confirming benefits… which would be impossible for somebody [with a] busy clinical practice to do,” while a financial counselor in California estimated spending at least 10 to 30 hours per patient verifying coverage.

Participants reported that health insurance plan FP benefit designs were problematic due to heterogeneity, lack of parity in coverage between FP services and other medical services, and lack of coverage of essential FP services. Benefit heterogeneity led to challenges in estimating out-of-pocket costs for patients; one administrator in New York said, “Depend[s] on if they have any deductibles, any type of out-of-pocket expense with the insurance. So there can definitely be a wide variation.”

Respondents also acknowledged the lack of parity between FP and other medical benefits; a financial counselor in California noted, “We have a lot of HMO [health maintenance organization] plans that… services for fertility are covered at a 50% coinsurance, where [for] their medical benefits, there’s no coinsurance.” Pharmacy benefits may be carved out from medical benefits and have separate annual or lifetime limits. Respondents reported confusion about which FP services were covered and frustration that standard services were sometimes not covered at all; a financial counselor in California said, “Usually they’re able to get coverage for the monitoring, so ultrasound portion, but it gets [trickier] to get coverage for the actual cryopreservation [freezing], which is the main point of the treatment.”

Fertility clinicians and financial counselors highlighted health insurance representatives’ lack of FP knowledge as a major barrier. Clinic staff reported educating insurance representatives on the FP benefit mandate and differences between infertility and FP benefits to circumvent outright denials for treatment; a financial counselor in California said, “I have encountered that currently most insurance representatives are not very familiar with iatrogenic [treatment-induced] infertility and how that’s different from standard infertility.”

Another highly ranked barrier was the time required to verify benefits, especially before cancer treatment begins; a clinician in Illinois said, “I think the struggle is if someone comes and sees you and her [chemotherapy] is starting in 3 weeks, we don’t have a luxury of time…. And some, understandably, patients don’t want to start unless they have clarity of coverage.” Clinics and patients often received conflicting and incorrect information from provider services and member services departments, with one clinic administrator in New York saying, “We’ve called the insurance, they are telling us that the patient doesn’t have any benefits, the patient has called on their own, and they’re being told they have full coverage.” Without verified benefits, patients drop out before seeing fertility specialists or undergoing FP services, as most clinics require full payment up-front. This up-front financial burden, time burden, and coverage uncertainty led to patients either not receiving recommended FP services or experiencing financial hardship.

Participants noted barriers related to claims systems not being updated to include FP procedure codes separate from infertility codes. For example, when FP diagnoses and services are hierarchically classified under infertility and not cancer treatment, coverage may be denied for covered FP services if a patient does not have coverage for infertility services; a financial counselor in California said, “They [insurance companies] can’t remove the fact that these codes are related to infertility… and so the insurance companies have a hard time disconnecting the two because in their system, they always come in as infertility procedure codes.”

The lack of reliable information available on insurer websites on covered FP services and in-network clinicians and facilities was also a barrier. Moreover, patients sometimes faced inadequate clinician and facility networks, including mismatches, where in-network clinicians did not practice at in-network facilities; a financial counselor in California said, “The hospital itself may be in-network, but a specialty doctor [who practices there] may not. So that’s where it gets complex for the patients.”

#### Clinic Level

The main barrier at the clinic level was frequent turnover of clinic financial staff. Smaller clinics described higher turnover, making it more difficult for well-trained staff to provide patients with appropriate financial counseling and support for claims and appeals; an administrator in Illinois said, “I tell somebody new coming in, it’s going to take you a good year to even understand all of the ins-and-outs of infertility or FP because everything is changing all the time. Technology is changing. Procedures are changing. Insurance plans are changing.” Larger clinics were more likely to have staff trained for different financial functions, such as benefit verification and authorization processes among insurers, patient financial counseling, and claims and appeals processes.

#### Patient Level

A key barrier described by participants was patients’ lack of knowledge of their covered benefits and how to find them. Respondents mentioned that it would be useful if FP benefits were clearly described in member handbooks, with one administrator in Illinois remarking that patients are “still somewhat confused because their plan benefit booklets [are]… so restricted in the information that they put there, that the patients still aren’t sure what’s being covered, what isn’t being covered.” In addition, financial counselors indicated there is a lack of financial resources available for patients without insurance coverage.

### Facilitators

#### Insurer Level

Two insurer-level facilitators were important to increasing access: (1) FP benefits being clearly described in members’ health insurance plan handbooks and (2) health insurance plans having an existing infertility benefit. When benefits are clearly described in member handbooks, it makes it easier to confirm benefit coverage and start treatment sooner. Additionally, when there are preexisting infertility benefits, FP benefits are easier to access because the infrastructure for providing coverage of fertility procedures exists.

#### Clinic Level

Participants described a number of facilitators related to clinic financial counselors’ activities, expertise, and contacts. Clinics with experienced staff dedicated to interacting with insurers reported more success with verifying benefits, receiving prior authorization requests, and receiving payment for covered services. Some clinics attributed their success to identifying an expert navigator that spans the range of clinical and financial support for FP; a financial navigator in Illinois said, “I think an individual like [the clinic’s administrator] is kind of this thing that keeps everything moving, just kind of an expert of all things within [our] population.” Clinics also cited the importance of identifying a specific, knowledgeable contact at the health insurer; a financial administrator in California said, “With [health plans], in particular, they’re impossible to get to in terms of getting to the person to talk to, literally impossible… Everyone that finally makes contact with a [health plan] representative, they keep that number like it’s gold.” Less important facilitators included generating a list of FP diagnosis and *Current Procedural Terminology* (CPT) codes and generating a templated letter to send to health insurers to cite medical necessity or appeal denied benefits or claims.

#### Patient Level

Financial counselors emphasized the importance of educating patients by providing a script for patients to follow with insurers to learn about their coverage specifics. One New York clinic administrator specified that “we send them in the right direction to say okay, if you’re calling for benefits, you want to make sure that you’re telling them specifically what you’re doing and then here are the *CPT* codes.” Assistance with navigation or materials for appeals, accessing loans and philanthropic resources, and transparency on FP service costs were lower-ranked patient support strategies.

## Discussion

Following FP mandate passage, clinics across different state mandate contexts adapted to the availability of insurance coverage for FP services and reported a range of challenges to secure coverage from patients’ health insurer, the most significant of which were time needed and health insurance navigation expertise. Findings inform clinic policies to support clinic and patient interactions with insurers, insurer policies to facilitate access, and state laws and regulatory actions to support compliance.

Building expertise on benefit verification, prior authorization, claims, appeals, and escalation processes for different health insurers were key facilitators for insurer-level barriers, which requires clinics to commit resources. To support patient-insurer interactions, patients can be provided with health insurance literacy tutorials, tip sheets for interacting with health insurers, benefit verification scripts, FP *International Statistical Classification of Diseases and Related Health Problems *and *Current Procedural Terminology* codes, provider identifiers and facilities identifiers for in-network status, and appeals letter templates. Clinics in all 3 states reported confusion between infertility and FP benefits as being a barrier to patient access, despite the existence of infertility benefit mandates in New York and Illinois. This suggests the importance of written information on FP benefit mandates and the distinction between FP and infertility services for clinics and patients to educate insurers and facilitate access. The effectiveness of these strategies to increase access to mandated benefits is not known and warrants future trials. In the interim, clinics can review, select, and implement relevant identified determinants to facilitate access to FP services.

At the insurer level, health plan handbooks with clear identification and description of FP benefits would save clinics and patients time spent on benefit verification. Among benefit designs, significant holes exist in comprehensive coverage of FP services, such that patients remain responsible for large out-of-pocket payments that go beyond copays. This points to the need to develop a model insurance benefit, based on scientific evidence, led by clinical organizations such as the American Society of Reproductive Medicine and American Society of Clinical Oncology. In 2008, the US Public Health Service produced a Clinical Practice Guideline on Treating Tobacco Use and Dependence.^17^ This guideline made specific, evidence-based recommendations related to insurance coverage and health system strategies to treat tobacco use and dependence. The development of a similar guideline for FP benefits could be useful for insurer policies on benefit design and state regulations for implementing mandates.

A major barrier preventing patients from accessing FP benefits is that approximately 54% of employers in the US provide self-funded health insurance that is regulated by federal law and not subject to state benefit mandates.^18^ Thus, increasing access to coverage would require passage of a federal FP benefit mandate requiring all employers and publicly funded programs to provide FP benefits. In 1998, the Women’s Health and Cancer Rights Act was passed at the federal level.^19^ This required any health plan that provided coverage for mastectomy to also cover reconstructive surgery. This legislation serves as a model for legislation to require all insurers who provide coverage for treatments that could impair fertility to also cover FP services with parity. A federal mandate would not only simplify the process of verifying benefits but also could potentially reduce racial and ethnic disparities in utilization of FP services by expanding the pool of those eligible for FP coverage across the US.^20^

This study adds to the previous research documenting challenges with implementing state benefit mandates for autism and mental health services.^21,22^ This literature similarly notes the challenge of verifying coverage and suggests creation of a federal database where patients and clinics can search if employer-sponsored coverage is self-funded.^21^ In addition, challenges related to lack of specificity of the mandate, high cost-sharing requirements, and network adequacy issues were also noted.^22^ The result of these barriers is increased administrative burden on patients, which has shown to be associated with a delay in receipt of care and may be especially burdensome for patients with urgent treatment needs, such as patients with newly diagnosed cancer.^23,24,25^

### Limitations

This study has limitations. We acknowledge that the generalizability of our findings may be limited by most of the participating clinics being from California (15 of 23 [65%]). With each wave of data collection, we began to note that similar themes were arising in clinics across the 3 different states. We conducted interviews until additional interviews in Illinois and New York failed to yield new insights. In addition, we conducted a sensitivity analysis that found that the ranking of barriers and facilitators for New York and Illinois were the same in the top, middle, and bottom tertiles with and without the inclusion of California clinics (eTables 2 and 3 in Supplement 1). Future work should quantify the impact that mandate structures across different states has on implementation and patient utilization. In addition, our team is embedded in a cancer center and focused this project on cancer-related FP. Therefore, understanding determinants of other medically indicated FP, eg, gene therapy for hematologic disease, may not be represented in this study. Finally, because we purposively sampled states based on heterogeneity of benefit mandate conditions, our findings should be transferable to other states outside of our sample.

## Conclusions

In this mixed-methods study of fertility and oncology clinic representatives, multiple barriers and facilitators were systematically identified to assist in improving implementation of FP benefit mandates. With increasing momentum for state and federal policies to expand coverage of FP services, delineating barriers and facilitators to patient access is central to increasing utilization. Guided by implementation science frameworks, this study systematically identified avenues for improving access to FP services. Results should inform future development of FP benefit mandate legislation and regulation, as well as implementation strategies for fertility clinics and insurers.

## Notes

Supplement 1. **eTable 1.** FP Legislation in Effect by January 1, 2020 (ie, the Start of the Study Period) **eTable 2.** Sensitivity Analysis of Identified Barriers to FP Services and Stakeholder Ranking of Perceived Level of Harm Including and Excluding California **eTable 3. **Sensitivity Analysis of Identified Facilitators to FP Services and Stakeholder Ranking of Perceived Level of Helpfulness Including and Excluding California

Supplement 2. Data Sharing Statement
