# Supplementary material for: Improving Implementation of Fertility Preservation Benefit Mandates
Source: JAMA Health Forum. 2025 Sep 12;6(9):e253166. doi: 10.1001/jamahealthforum.2025.3166 (PMC12432631; doi:10.1001/jamahealthforum.2025.3166)
Supplement: Supplement 1. — eTable 1. FP Legislation in Effect by January 1, 2020 (ie, the Start of the Study Period) eTable 2. Sensitivity Analysis of Identified Barriers to FP Services and Stakeholder Ranking of Perceived Level of Harm Including and Excluding California eTable 3. Sensitivity Analysis of Identified Facilitators to FP Services and Stakeholder Ranking of Perceived Level of Helpfulness Including and Excluding California [file jamahealthforum-e253166-s001.pdf]

Supplemental Online Content

McMenamin SB, Kaiser BN, Ortega RF, et al. Improving implementation of fertility preservation benefit mandates. *JAMA Health Forum*. 2025;6(9):e253166. doi:10.1001/jamahealthforum.2025.3166

eTable 1. FP Legislation in Effect by January 1, 2020 (ie, the Start of the Study Period)
eTable 2. Sensitivity Analysis of Identified Barriers to FP Services and Stakeholder Ranking of Perceived Level of Harm Including and Excluding California
eTable 3. Sensitivity Analysis of Identified Facilitators to FP Services and Stakeholder Ranking of Perceived Level of Helpfulness Including and Excluding California

This supplementary material has been provided by the authors to give readers additional information about their work.

© 2025 McMenamin SB et al. *JAMA Health Forum*.

eTable 1. FP Legislation in Effect by January 1, 2020 (ie, the Start of the Study Period)

State	FP Mandate Effective Date	Pre-Existing IVF Mandate[1]	Mandate Population[2]	Policy Coverage Specificity[3]	Fertility Medication Coverage Specified
**CA**	**10/12/19**	**No**	**Commercial**	**Vague**	**Not specified**
CT	1/1/18	Yes	Commercial	Somewhat Specific	Not specified
DE	6/30/18	No, concurrent	Commercial	Specific	Specified
**IL**	**1/1/19**	**Yes**	**Commercial; Medicaid**	**Somewhat Specific**	**Not specified**
MD	1/1/19	Yes	Commercial	Somewhat Specific	Specified
NH	1/1/20	No	Commercial	Specific	Specified
**NY**	**1/1/20**	**No, concurrent**	**Commercial**	**Specific**	**Specified**
RI	7/5/18	Yes	Commercial	Vague	Not specified

Source: Ortega et al., 2021

Notes:[1] "No" indicates that there was not an infertility mandate in place at the time the FP mandate was passed, "Yes" indicates that there was a pre-existing infertility mandate, and "No, concurrent" indicates that the IVF and FP mandates were passed at the same time [2] All states offer coverage for patients at risk of infertility from any medical treatment (i.e. iatrogenic infertility) [3] Data taken from Table 2 in Ortega et al. The level of specificity was calculated by the number of FP services that were specifically mentioned in the legislation or regulator guidance: Vague (0-2 FP services), Somewhat specific (3-5 FP services), and Specific (6-9 FP services).

© 2025 McMenamin SB et al. *JAMA Health Forum*.

eTable 2. Sensitivity Analysis of Identified Barriers to FP Services and Stakeholder Ranking of Perceived Level of Harm Including and Excluding California

	California Excluded	California Included
Patient lacks knowledge on existence, access, and content of health plan handbook or Fertility preservation benefits	1	2
Not all health insurance is subject to the fertility preservation mandate (examples: Medicaid in CA and NY; health insurance from an employer not headquartered in state)	2	4
Calling member services or provider services is time consuming & requires both the patient and clinic	3	1
Fertility preservation benefit designs (what is covered) vary greatly, even within the same insurer	4	3
Different insurers have different benefits verification procedures or none at all	5	7
Fertility preservation services diagnostic and CPT codes are not in the insurer benefit verification, preauthorization or claims system(s)	6	8
Frequent turnover of clinic financial staff	7	5
Insurer member/provider services confuses fertility preservation with infertility services	8	6
On insurer website, search lacks specificity (example: infertility finds general ob/gyn instead of reproductive endocrinologists) or not updated for Fertility preservation providers (example: retired physicians) and facilities (example: no facilities under "IVF")	9	11
Application to loans and philanthropic resources are difficult to find and complete	10	9
Fertility preservation providers and the facilities where they work are not both in network within the same health plan	11	10

© 2025 McMenamin SB et al. *JAMA Health Forum*.

eTable 3. Sensitivity Analysis of Identified Facilitators to FP Services and Stakeholder Ranking of Perceived Level of Helpfulness Including and Excluding California

	California Excluded	California Included
Clinic financial counselor guides patients on how to verify fertility preservation benefits (with insurer member services or employer human resources).	2	1
Fertility preservation benefit is clearly described in a patient's health plan handbook.	1	2
Health plans (example: Anthem Plan A) have an pre-existing IVF benefit for infertility.	4	3
Clinic financial counselor has contacts they can directly reach at insurer provider services for benefit verification, prior authorization, claims or appeals.	3	4
Clinic financial counselor has the expertise to escalate to insurer supervisors when needed during benefit verification, prior authorization, claims or appeals.	6	4
Clinic financial counselor has expertise on benefit verification processes among different insurers.	7	6
Having a dedicated clinical financial counselor.	5	7
Clinic financial counselor educates insurers about Fertility preservation law during benefit verification, prior authorization, claims or appeals.	8	8
Patient's employer human resources is knowledgeable about fertility preservation benefit and how to access it.	9	9
Easy access to a list of fertility preservation diagnosis and CPT codes.	10	10
Templated letter of medical necessity for fertility preservation from the provider to the insurer that includes statement of the state law requiring coverage for Fertility preservation services.	12	11
Clinic financial team educates patient how to submit appeals for fertility preservation services.	11	12
Clinic financial team guides patient on accessing loans and philanthropic resources for fertility preservation.	13	12
Templated appeal documents for fertility preservation services provided to patient.	15	14
Patient facing materials for clinic financial counselor to discuss fertility preservation service costs.	14	15

© 2025 McMenamin SB et al. *JAMA Health Forum*.
