# Supplementary material for: Improving Implementation of Fertility Preservation Benefit Mandates
Source: JAMA Health Forum. 2025 Sep 12;6(9):e253166. doi: 10.1001/jamahealthforum.2025.3166 (PMC12432631; doi:10.1001/jamahealthforum.2025.3166)
Supplement: Supplement 2. — Data Sharing Statement [file jamahealthforum-e253166-s002.pdf]

Data Sharing Statement

McMenamin. Improving Implementation of Fertility Preservation Benefit Mandates. *JAMA Health Forum*. Published September 12, 2025. doi:10.1001/jamahealthforum.2025.3166

Data
Data available: No
